# *In silico* comparisons of lipid-related genes between *Mycobacterium tuberculosis* and BCG vaccine strains

**DOI:** 10.1590/1678-4685-GMB-2021-0024

**Published:** 2021-10-22

**Authors:** Alice Sarno, Julia Bitencourt, Adriano Queiroz, Sergio Arruda

**Affiliations:** 1 Fundação Oswaldo Cruz, Instituto Gonçalo Moniz, Laboratório Avançado em Saúde Pública, Salvador, BA, Brazil.; 2 Universidade Federal da Bahia, Salvador, BA, Brazil.; 3 Universidade do Estado da Bahia, Salvador, BA, Brazil.

**Keywords:** Genome comparison, BCG, lipid, cell-wall, tuberculosis

## Abstract

Despite highly variable efficacy, BCG (Bacillus Calmette-Guérin) is the only vaccine available to prevent the tuberculosis (TB). Genomic heterogeneity between attenuated BCG strains and virulent *Mycobacterium tuberculosis* might help to explain this vaccine’s impaired capacity to induce long-term protection. Here, we investigate the lipid-related genes absent in attenuated BCG strains in order to correlate changes in both lipid metabolism and cell-wall lipid content to vaccine impairment. Whole genome sequences of *M. tuberculosis* H37Rv and the six most used BCG strains worldwide were aligned and the absent regions functionally categorized. Genomes of the BCG strains showed a total of 14 non-homologous lipid-related genes, including those belonging to *mce3* operon, as well as the gene *echaA1*, which encodes an enoyl-CoA hydratase, and the genes encoding phospholipases PlcA, PlcB and PlcC. Taken together, the depletion of these *M. tuberculosis* H37Rv genomic regions were associated with marked alterations in lipid-related genes of BCG strains. Such alterations may indicate a dormant-like state and can be determining factors to the vaccine’s inability to induce long-term protection. These lipids can be further evaluated as an adjuvant to boost the current BCG-based vaccine.

## Introduction

Tuberculosis (TB), caused by *Mycobacterium tuberculosis*, is a leading cause of death worldwide: In 2019 alone, 1.2 million deaths and 10 million new cases were reported. About a quarter of the world population is estimated to harbor latent TB infection and are therefore at risk of developing active disease (WHO, 2020). 

BCG (Bacillus Calmette-Guérin), a live-attenuated strain of *Mycobacterium bovis*, is currently the only vaccine available to prevent TB, typically administrated in endemic countries or in populations at high risk of infection (Cernuschi et al., 2018). Currently, six strains account for more than 90% of the vaccines in use worldwide: early strains BCG Moreau, BCG Russian and BCG Tokyo, and late strains BCG Danish, BCG Glaxo and BCG Pasteur (WHO, 2012, 2017). Despite its capacity to protect against disease progression and disseminated forms of TB, the efficacy of BCG against pulmonary TB in adult populations varies from 0% to 80% (Mangtani et al., 2014; Roy et al., 2014). 

One of the hypotheses to explain the variable protectiveness of BCG posits the genomic heterogeneity between vaccine and virulent *M. bovis* and *M. tuberculosis* strains (Behr 2002; Liu et al., 2009; Angelidou et al., 2020). Since it was first obtained and distributed, BCG has accumulated large sequence polymorphisms and has lost several virulence factor genes, including deletion of the region RD1, which encodes antigenic proteins ESAT-6 and CFP-10 (Mahairas et al., 1996; Lewis et al., 2003). However, the expression of RD1 in recombinant BCG does not result in a complete restoration of protection against TB, which could indicate that other mechanisms may be involved in virulence (Pym et al., 2003). 

The genomic differences between BCG strains and virulent *M. bovis* and *M. tuberculosis,* as well as the remodeling of protein complexes, have been comprehensively explored through phylogenetic analysis (Brosch et al., 2007; Zhang et al., 2013; Abdallah et al., 2015). However, the impact of genomic heterogeneity on virulence factors related to mycobacteria cell-wall lipid content and lipid metabolism has received less attention (Abdallah *et al.,* 2015). Discrepancies in lipid species in the cell walls of virulent and attenuated strains of mycobacteria might play a key role in host-pathogen interaction (Guenin-Macé et al., 2009; Queiroz and Riley 2017; Mishra et al., 2019). In BCG, genome polymorphisms and the absence of specific cell-wall lipid components have resulted in less-virulent strains that induce a restrained pro-inflammatory immune response and limit BCG-mediated T cell protection, with diminished immunological activity (Hayashi et al., 2009; Tran et al., 2016; Zhang *et al.,* 2016). 

Here we compared the whole genome sequences of *M. tuberculosis* H37Rv and the six BCG strains more frequently used worldwide in an attempt to identify genomic differences related to lipid content and metabolism. By this approach, we established a comprehensive list of lipid-related genes absent in these BCG strains, in which the codified molecules may contribute to improve the BCG vaccines currently in circulation. 

## Material and Methods

### Whole genome sequence selection

The following whole genome sequences stored on GenBank were compared *in silico*: *M. tuberculosis* H37Rv (accession number NC_000962.3), early strains *M. bovis* BCG Moreau RDJ (accession number AM412059.2), *M. bovis* BCG Russian 368 (accession number CP009243.1) and *M. bovis* BCG Tokyo 172 (accession number AP010918.1), and late strains *M. bovis* BCG Danish 1331 (accession number CP039850.1), *M. bovis* BCG Glaxo (accession number NZ_CUWJ01000001.1) and *M. bovis* BCG Pasteur 1173P2 (accession number AM408590.1). The six BCG strains were selected for comparison with *M. tuberculosis* H37Rv, since these account for more than 90% of the vaccines in use worldwide.

### Determination of homologous and non-homologous regions among sequences

Mauve software (Darling et al., 2010) was used to align, identify and characterize homologous and non-homologous regions among the whole genomes. Regions were considered homologous if percent identity was > 60% and query cover was > 70%. After alignment, the gene annotations for homologous and non-homologous regions were obtained and exported as comma-separated values for further analysis. The number and percentage of homologous and non-homologous regions between each BCG strain and the *M. tuberculosis* H37Rv sequence were compared to measure the similarity among genomes. Finally, the gene annotations for non-homologous regions in each BCG strain were confirmed by BLASTN searches in the NCBI database (Morgulis et al., 2008).

### Functional category determination of non-homologous regions of early and late strains of BCG

The gene annotations in non-homologous regions confirmed by BLASTN searches were functionally categorized using the Mycobrowser database (Kapopoulou et al., 2011). Early (BCG Moreau RDJ, BCG Russian and BCG Tokyo 172) and late (BCG Danish 1331, BCG Glaxo 1077 and BCG Pasteur 1173P2) BCG strains were compared to the *M. tuberculosis* H37Rv genome.

## Results

### Similarities among homologous and non-homologous regions

**Figure 1 ‒:**
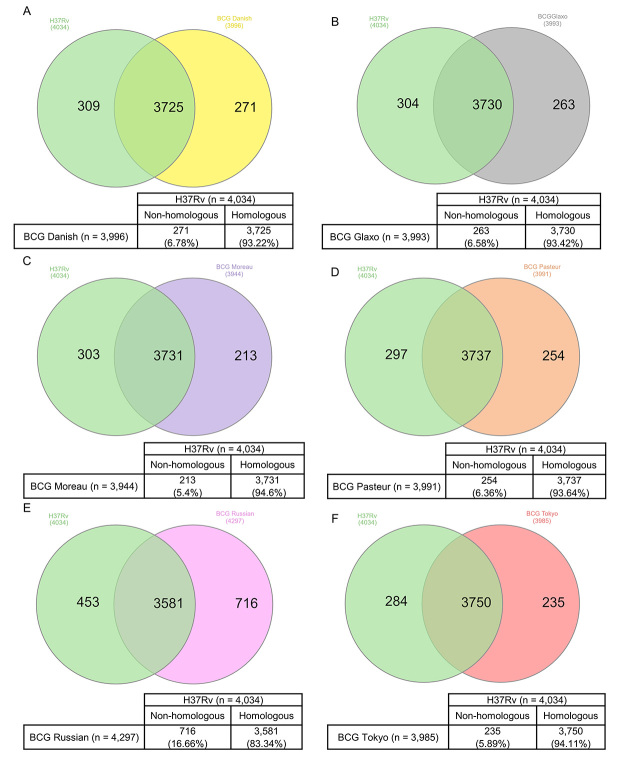
Homologous and non-homologous regions between *M. tuberculosis* H37Rv, early BCG strains and late BCG strains genome sequences. Venn diagrams showing overlap between and the total number of homologous and non-homologous regions across (A) *M. tuberculosis* H37Rv and BCG Danish, (B) *M. tuberculosis* H37Rv and BCG Glaxo, (C) *M. tuberculosis* H37Rv and BCG Moreau, (D) *M. tuberculosis* H37Rv and BCG Pasteur, (E) *M. tuberculosis* H37Rv and BCG Russian and (F) *M. tuberculosis* H37Rv and BCG Tokyo.

Sequence alignment was performed using Mauve software to investigate differences and similarities between the *M. tuberculosis* H37Rv and BCG strains genomes and to better visualize homology among the studied genomes. 4,034 genomic regions were identified in the *M. tuberculosis* H37Rv genome, 3,996 in BCG Danish, 3,993 in BCG Glaxo, 3,944 in BCG Moreau, 3,991 in BCG Pasteur, 4,297 in BCG Russian and 3,985 in BCG Tokyo (Figure 1). 

The overlap between and the total number of homologous and non-homologous regions across *M. tuberculosis* H37Rv and all six BCG genome sequences, represented as Venn diagrams, are illustrated in Figure 1. As expected, high homogeneity was observed between most BCG strains and *M. tuberculosis* H37Rv, with up to 94.6% of homologous regions identified in the BCG Moreau genome. BCG Russian was the strain with the greater number of non-homologous regions (16.7%), when compared to *M. tuberculosis* H37Rv. Together, the data shows a comparable genomic heterogeneity between each strain and *M. tuberculosis* H37Rv, as well as the overall similarity among attenuated vaccine strains (Figure S1).

### Functional category identification of genes in non-homologous regions

**Figure 2 ‒:**
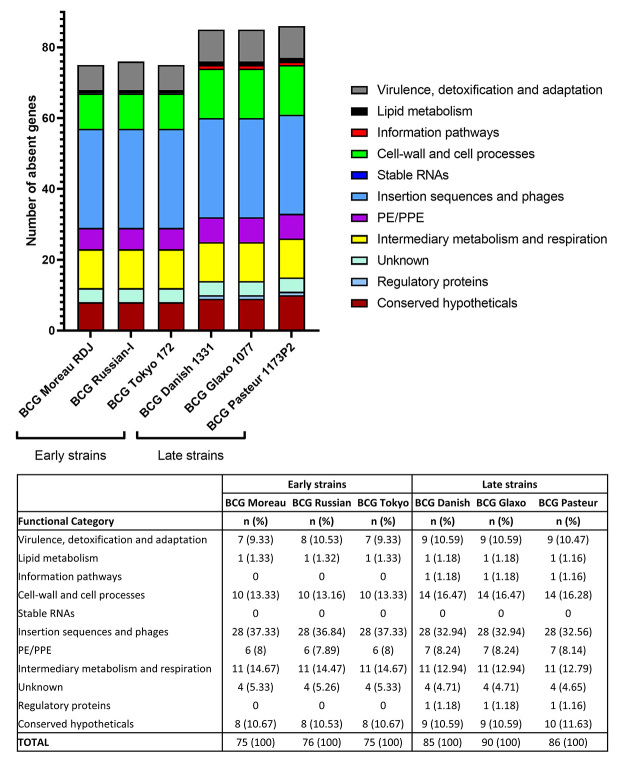
Functional categories of non-homologous genes in early and late BCG strains compared to *M. tuberculosis* H37Rv. n (%): number and percentage of non-homologous genes in each functional category relative to the total number of non-homologous genes per genome.

The genes in non-homologous regions of all BCG strains identified in the alignment were confirmed by BLASTN searches and grouped according to functional category using the Mycobrowser database (Figure 2). The distribution of non-homologous regions **-** with no BLASTN similarity - in each functional category was similar among strains from the same phylogenetic groups: early strains (BCG Moreau, BCG Russian and BCG Tokyo) and late strains (BCG Danish, BCG Glaxo and BCG Pasteur).

Most of the identified non-homologous regions were associated with functional category “insertion sequences and phages”. In comparison to *M. tuberculosis* H37Rv, the number of non-homologous genes in this category was 28 among all BCG strains, representing between 31.11% and 37.33% of all non-homologous regions. In the early strains, the next most common identified category was “intermediary metabolism and respiration”, with 11 (between 14.1% and 14.67%) non-homologous regions, followed by “cell-wall and cell processes”, with 10 (between 12.82% and 13.33%) non-homologous regions. In the late strains, the opposite was identified: “cell-wall and cell processes” was the second most common identified category, with 14 (between 15.56% and 16.28%) non-homologous regions, followed by “intermediary metabolism and respiration”, with 11 (between 12.22% and 12.79%) non-homologous regions. Other categories also associated with non-homologous regions included “conserved hypotheticals” and “virulence, detoxification and adaptation”.

Highlighted in Table 1 are the 14 lipid-related genes absent in all six BCG vaccine strains most commonly used worldwide, when compared to *M. tuberculosis* H37Rv. The genes are associated with the functional categories “cell-wall and cell processes” (4), “virulence, detoxification and adaptation” (6), “lipid metabolism” (1) and “intermediary metabolism and respiration” (3). The complete list of absent genes, in all functional categories, is described in Table S1.

**Table 1 ‒:** **M. tuberculosis** H37Rv lipid-related genes corresponding to non-homologous regions in all six BCG strains.

Functional category	H37Rv gene locus	Gene product
Cell wall and cell processes	Rv1970	mce3E
	Rv1972-Rv1974	Mce associated membrane proteins
Virulence, detoxification, adaptation	Rv1965-Rv1969	YrbE3B, Mce3A, Mce3B, Mce3C and Mce3D
	Rv1971	Mce3F
Lipid Metabolism	Rv0222	Enoyl-CoA hydratase (EchA1)
Intermediary metabolism and respiration	Rv2349c-Rv2351c	Phospholipases C (PlcC, PlcB and PlcA)

Ten of these 14 genes belonged to the *mce3* operon: four were in the “cell wall and cell processes” category (Rv1970 and Rv1972 to Rv1974) and six (Rv1965 to Rv1969 and Rv1971) in the “virulence, detoxification, adaptation” category. The gene encoding enoyl-CoA hydratase (Rv0222), which is part of the fatty acid degradation metabolism, was categorized as “lipid metabolism”. Finally, three genes encoding phospholipases PlcC, PlcB and PlcA (Rv2349c to Rv2351c, respectively), related to lipid metabolism, were included in the “intermediary metabolism and respiration” category. 

Differences between *M. tuberculosis* H37Rv and BCG strains, previously established in the literature, were also verified. The absence of the five genes encoding the ESAT-6 secretion system-1 (ESX-1) in all six strains: Rv3874 (*esxB*), Rv3875 (*esxA*), Rv3876 (*espI*), Rv3877 (*eccD1*) and Rv3878 (*espJ*), comprised the “cell wall and cell processes” category (Table S1) (Hsu et al., 2003; Lewis et al., 2003). In addition, the mutation in the Rv2930 (*fadD26*) and Rv2931 (*ppsA*) loci, which impairs the biosynthesis of phthiocerol dimycocerosates (PDIMs) and phenolic glycolipids (PGLs) in BCG Moreau (Chen et al., 2007; Leung et al., 2008), was verified (data not shown).

## Discussion

The present study identified and categorized a comprehensive list of absent lipid-related genes shared by the most used BGC strains worldwide compared to the *M. tuberculosis* H37Rv genome. The *M. tuberculosis* H37Rv genome, and not *M. bovis*, was used as reference genomic sequence to assure comparison between vaccine strains and the best curated sequence of the causative agent of TB. The cell wall lipid content of mycobacteria plays an important role in the pathogen-host interaction and inflammation (Forrellad et al., 2013; Dulberger et al., 2020; Petrilli et al., 2020). Thus, these genes can be further explored as common targets for virulence and efficacy improvement of the BCG vaccine currently in circulation.

Since the sequencing of the *M. tuberculosis* H37Rv genome in 1998, the *in silico* comparisons of genus, species and isolates have resulted in abundant data of mycobacterial sequences (Cole et al., 1998, 2001; Fraser et al., 2000; Gordon *et al.,* 2001). Furthermore, comparative genomic analyses have supported many molecular based hypotheses regarding the impact of protein virulence factors on the protection induced by BCG strains (Behr et al., 1999; Lewis et al., 2003; Sherman et al., 2004; Zhang et al., 2013). However, the role of mycobacterial cell-wall content and lipid metabolism on virulence has received less attention, with analyses often limited in number of lipid antigens and BCG strains included, as well as comparisons with *M. bovis* sequences (Rhoades et al., 2005; Layre et al., 2014; Abdallah et al., 2015; Tran et al., 2016; Gonzalo-Asensio et al., 2017; Jia et al., 2017; Wright et al., 2017). 

Genes at loci Rv1965 to Rv1974, which encode proteins from the *mce3* operon, were found to be absent in all BCG strains, accounting for 10 of the 14 non-homologous genes identified. The *mce3* operon is an important virulence factor, since *M. tuberculosis* strains disrupted on this operon displayed longer survival and lower colony-forming units (CFU) in mice and guinea pig models (Gioffré et al., 2005; Obregón-Henao et al., 2011). Proteins Mce3A (Rv1966), Mce3D (Rv1969) and Mce3E (Rv1970) also induced antibody response serum samples from TB patients (Ahmad et al., 2004). Similar to other *mce* operons, the products of *mce3* has been shown to affect the internalization process of mycobacteria (El-Shazly et al., 2007) and are possibly involved in cholesterol and fatty acids transport across the cell wall (Pandey and Sassetti 2008; Mohn et al., 2008; Perkowski et al., 2016). Interestingly, this intake of fatty acids seems to be greatly reduced in the BCG strains that do not produce PDIM (Nazarova et al., 2017), namely BCG Moreau, Tokyo and Glaxo.

A protein involved in the degradation of fatty acid was also found to be absent in all BCG strains compared to *M. tuberculosis* H37Rv. The gene *echaA1* (Rv0222) encodes an enoyl-CoA hydratase (EchA1) involved in energy production via β-oxidation, essential for mycobacterial survival and adaptation in environments with distinct fatty acids as the only carbon sources (Muñoz-Elías and McKinney 2006; Srivastava et al., 2015). Despite the gene redundancy involved in five pathways of β-oxidation, EchaA1 is secreted to the host cytosol and impairs the production of pro-inflammatory cytokines, by inhibiting TRAF6 (tumor necrosis factor (TNF)-receptor-associated factor 6) activation (Wang et al., 2020). 

With regard to synthesis of glycerolipids, phospholipase C (PlcC) (Rv2349c), PlcB (Rv2350c) and PlcA (Rv2351c) were absent in all BCG strains. These enzymes facilitate the hydrolysis of phosphatidylcholine, phosphatidylethanolamine (PE) and phosphatidylglycerol (PG) to produce diacylglycerol (DAG) (Srinivas et al., 2008) and their absence has been associated with reduced CFU in mice (Raynaud et al., 2002). Furthermore, pre-existing DAG is used for production of triacylglycerol (TAG), which is essential for the survival of *M. tuberculosis* in the host (Garton et al., 2008). 

Together, these 14 non-homologous genes may signal a lipid-dependent dormant-like state in all six BCG strains. The absence of *mce3* and *echa1* indicates an overall decline of cholesterol and fatty acid intake in BCG, that could result in lower carbon sources for lipid and energy production. In addition, the absence of *plcC*, *plcB* and *plcA* seems to be associated with lower levels of lipids upstream of DAG and higher levels of TAG. This condition has been previously described in BCG Pasteur (Layre et al., 2011, 2014) and related to long-term dormancy in *M. tuberculosis* (Daniel et al., 2004; Galagan et al., 2013). Therefore, while the lipids increased in level in *M. tuberculosis* H37Rv (such as PE, PG and trehalose-containing lipids) induce a more pro-inflammatory immune response, the accumulation of TAG could be favoring a dormant state in BCG strains.

The identification and study of genes related to cell-wall lipid content and lipid metabolism in BCG strains can contribute to elucidating the impact of attenuation on vaccine virulence and protection efficacy. We suggest that the *M. tuberculosis* lipid-related genes and its products that are absent in BCG should be explored as adjuvants alongside new vaccine candidates due to their capacity to enhance immune response.

## Supplementary material

The following online material is available for this article:

Table S1 - Complete list of H37Rv lipid-related genes corresponding to non-homologous regions in BCG-Moreau, -Danish, -Glaxo, -Pasteur, -Russian or -Tokyo.

Figure S1 - Homologous and non-homologous regions between *M. tuberculosis* H37Rv, early BCG strains and late BCG strains genome sequences.
