# Supplementary material for: In silico comparisons of lipid-related genes between Mycobacterium tuberculosis and BCG vaccine strains
Source: Genet Mol Biol. 2021 Oct 22;44(4):e20210024. doi: 10.1590/1678-4685-GMB-2021-0024 (PMC8547388; doi:10.1590/1678-4685-GMB-2021-0024)
Supplement: Table S1 - [file 1415-4757-GMB-44-4-e20210024-s1.pdf]

Genetics and Molecular Biology
https://doi.org/10.1590/1678-4685-GMB-2021-0024

Suplementary Material to "*In silico* comparisons of lipid-related genes between *Mycobacterium tuberculosis* and BCG vaccine strains"

Table S1 - Complete list of H37Rv lipid-related genes corresponding to non-homologous regions in BCG-Moreau, -Danish, -Glaxo, -Pasteur, -Russian or -Tokyo.

Functional category	H37Rv gene locus	Gene product
Virulence, detoxification and adaptation	Rv1965	Integral membrane protein YrbE3B
	Rv1966	Mce-family protein Mce3A
	Rv1967	Mce-family protein Mce3B
	Rv1968	Mce-family protein Mce3C
	Rv1969	Mce-family protein Mce3D
	Rv1971	Mce-family protein Mce3F
	Rv1982c[a]	Toxin VapC36
	Rv1982A[a]	Antitoxin VapB36
	Rv3617	Epoxide hydrolase EphA
	Rv3697A[b]	Antitoxin VapB48
Lipid metabolism	Rv0222	Enoyl-CoA hydratase (EchA1)
Information pathways	Rv1981c[a]	Ribonucleoside-diphosphate reductase (NrdF1)
Cell-wall and cell processes	Rv1508c	Probable membrane protein
	Rv1970	Mce3E
	Rv1972-Rv1974	Mce associated membrane protein
	Rv1980c[a]	Immunogenic protein Mpt64
	Rv1984c[a]	Cutinase precursor CFP21
	Rv1986[a]	Conserved integral membrane protein

Functional category	H37Rv gene locus	Gene product
	Rv1987[a]	Chitinase
	Rv3874	Antigen EsxB (CFP10)
	Rv3875	Antigen EsxA (ESAT-6)
	Rv3876	ESX-1 secretion-associated protein (EspI)
	Rv3877	ESX conserved component (EccD1)
	Rv3878	ESX-1 secretion-associated (EspJ)
Insertion sequences and phages	Rv1573-Rv1586c	Probable PhiRv1 phage protein
	Rv2646	Integrase
	Rv2647	Hypothetical protein
	Rv2650c-Rv2659c	Possible PhiRv2 prophage protein
	Rv3427c-Rv3428c	Possible transposase
PE/PPE	Rv1983[a]	PE-PGRS family protein (PE_PGRS35)
	Rv2352c	PPE family protein (PPE38)
	Rv3621c	PPE family protein (PPE65)
	Rv3622c	PE family protein (PE32)
	Rv3739c	PPE family protein (PPE67)
	Rv3872	PE family-related protein (PE35)
	Rv3873	PPE family protein (PPE68)
Intermediary metabolism and respiration	Rv1256c	Cytochrome P450 130 (Cyp130)
	Rv1511	GDP-D-mannose dehydratase (GmdA)
	Rv1512	Nucleotide-sugar epimerase (EpiA)
	Rv1516c	Sugar transferase
	Rv2073c	Shortchain dehydrogenase
	Rv2074	Possible pyridoxamine 5'-phosphate oxidase (PNP/PMP oxidase)
	Rv2349c	Probable phospholipase C 3 (PlcC)
	Rv2350c	Membrane-associated phospholipase C 2 (PlcB)
	Rv2351c	Membrane-associated phospholipase C 1 (PlcA)
	Rv3119	Molybdenum cofactor biosynthesis protein E (MoaE1)
	Rv3618	Possible monooxygenase

Functional category	H37Rv gene locus	Gene product
Unknown	Rv1507A	Hypothetical protein
	Rv1509	Hypothetical protein
	Rv2348c	Hypothetical protein
	Rv2645	Hypothetical protein
Regulatory proteins	Rv1985c[a]	Probable transcriptional regulatory protein (probably LysR-family)
Conserved hypotheticals	Rv1507c-Rv1508A	Conserved protein
	Rv1513-Rv1515c	Conserved protein
	Rv1769-Rv1770[c]	Conserved protein
	Rv1810[d]	Conserved protein
	Rv1975-Rv1976c	Conserved hypothetical protein
	Rv3120	Conserved hypothetical protein

[a]No BLASTN similarity in late strains (BCG Danish, BCG Glaxo and BCG Pasteur). [b]No BLASTN similarity in BCG Russia. [c]No BLASTN similarity in BCG Pasteur. [d]No BLASTN similarity in BCG Danish and BCG Glaxo.
