# Supplementary material for: In silico comparisons of lipid-related genes between Mycobacterium tuberculosis and BCG vaccine strains
Source: Genet Mol Biol. 2021 Oct 22;44(4):e20210024. doi: 10.1590/1678-4685-GMB-2021-0024 (PMC8547388; doi:10.1590/1678-4685-GMB-2021-0024)
Supplement: Figure S1 - [file 1415-4757-GMB-44-4-e20210024-s2.pdf]

Genetics and Molecular Biology
https://doi.org/10.1590/1678-4685-GMB-2021-0024

Suplementary Material to "*In silico* comparisons of lipid-related genes between *Mycobacterium tuberculosis* and BCG vaccine strains"

(a)

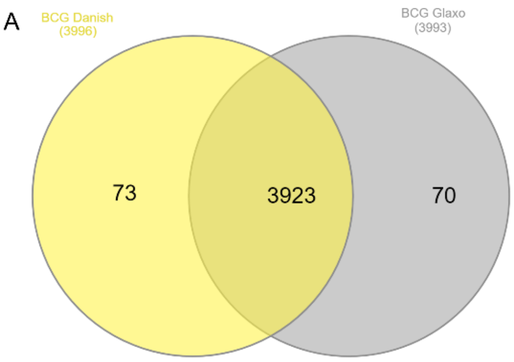

	BCG Danish (n = 3,996)	
	Non-homologous	Homologous
BCG Glaxo (n = 3,993)	73 (1.82%)	3,923 (98.18%)

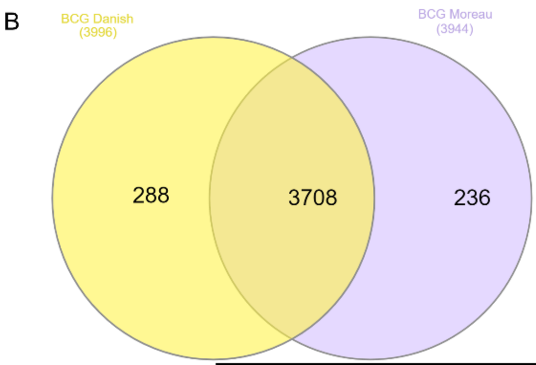

	BCG Danish (n = 3,996)	
	Non-homologous	Homologous
BCG Moreau (n = 3,944)	288 (7.2%)	3,708 (92.8%)

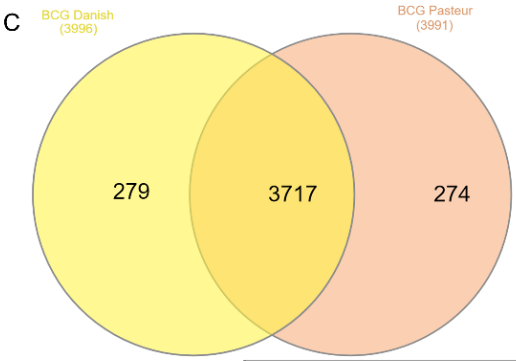

	BCG Danish (n = 3,996)	
	Non-homologous	Homologous
BCG Pasteur (n = 3,991)	279 (6.98%)	3,717 (93.02%)

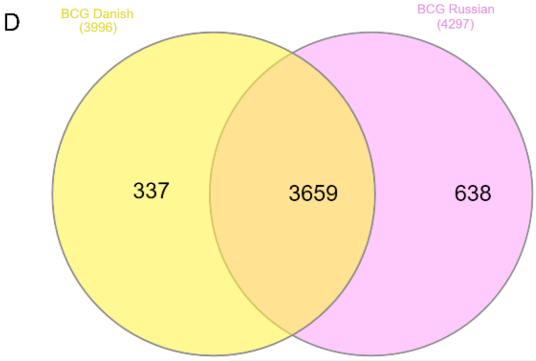

	BCG Danish (n = 3,996)	
	Non-homologous	Homologous
BCG Russian (n = 4,297)	337 (8.43%)	3,659 (91.57%)

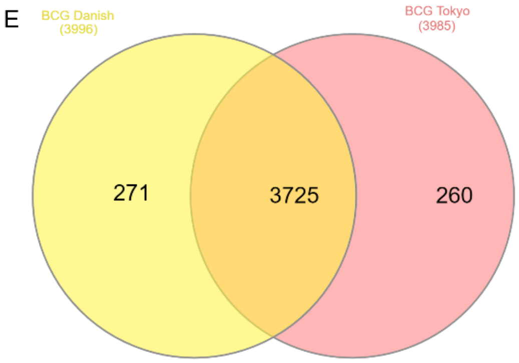

	BCG Danish (n = 3,996)	
	Non-homologous	Homologous
BCG Tokyo (n = 3,985)	271 (6.78%)	3,725 (93.22%)

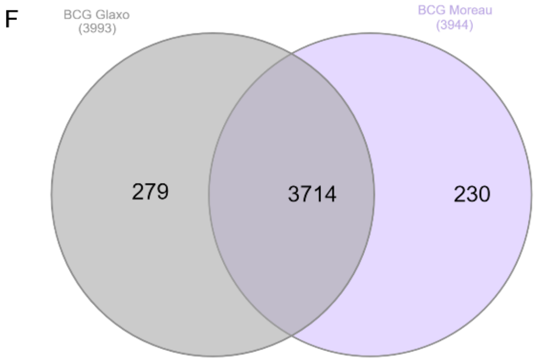

	BCG Glaxo (n = 3,993)	
	Non-homologous	Homologous
BCG Moreau (n = 3,944)	279 (6.98%)	3,714 (93.02%)

(b)

A

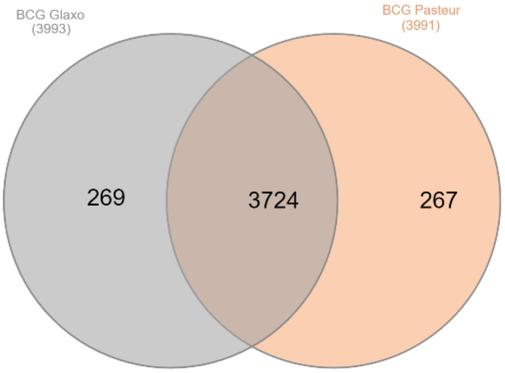

	BCG Glaxo (n = 3,993)	
	Non-homologous	Homologous
BCG Pasteur (n = 3,991)	269 (6.73%)	3,724 (93.27%)

B

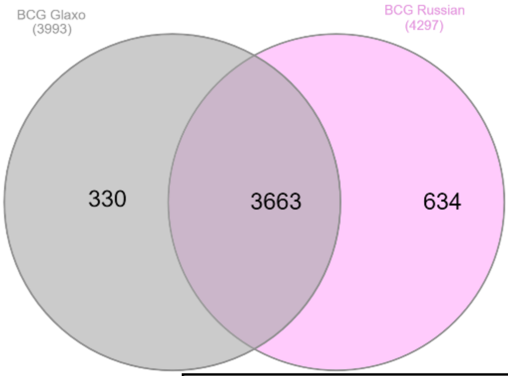

	BCG Glaxo (n = 3,993)	
	Non-homologous	Homologous
BCG Russian (n = 4,297)	330 (8.26%)	3,663 (91.74%)

C

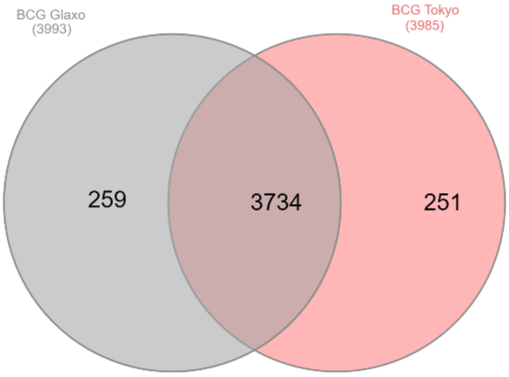

	BCG Glaxo (n = 3,993)	
	Non-homologous	Homologous
BCG Tokyo (n = 3,985)	259 (6.48%)	3,734 (93.52%)

D

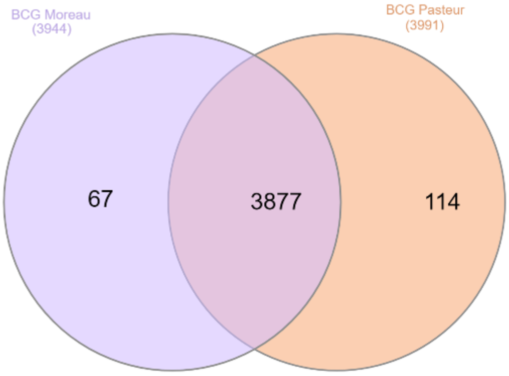

	BCG Moreau (n = 3,944)	
	Non-homologous	Homologous
BCG Pasteur (n = 3,991)	67 (1.69%)	3,877 (98.31%)

E

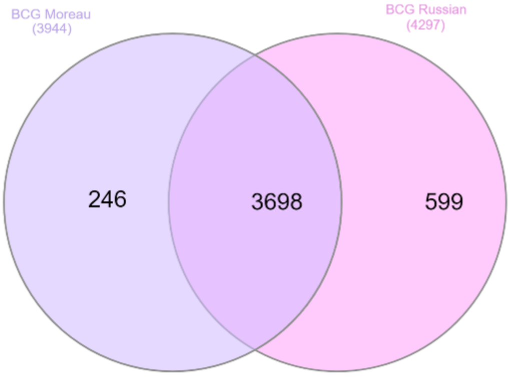

	BCG Moreau (n = 3,944)	
	Non-homologous	Homologous
BCG Russian (n = 4,297)	246 (6.23%)	3,698 (93.77%)

F

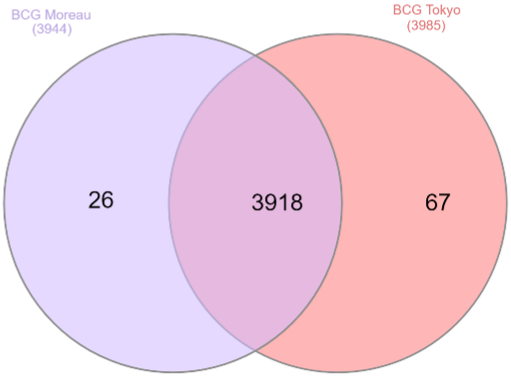

	BCG Moreau (n = 3,944)	
	Non-homologous	Homologous
BCG Tokyo (n = 3,985)	26 (0.65%)	3,918 (99.35%)

(c)

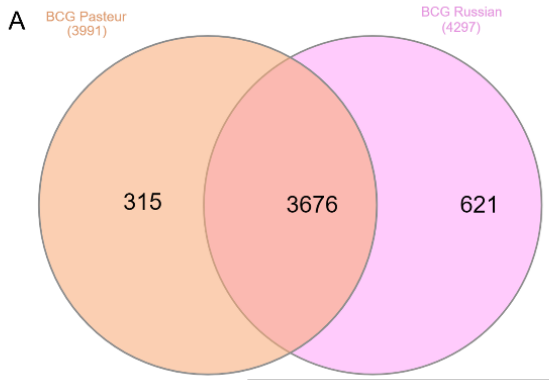

	BCG Pasteur (n = 3,991)	
	Non-homologous	Homologous
BCG Russian (n = 4,297)	315 (7.89%)	3,676 (92.11%)

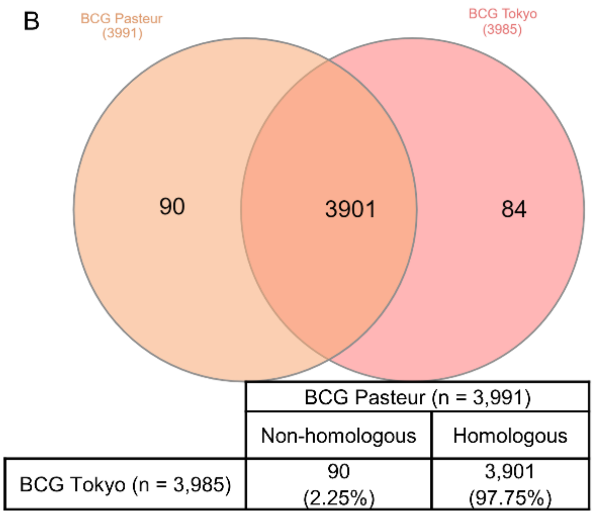

	BCG Pasteur (n = 3,991)	
	Non-homologous	Homologous
BCG Tokyo (n = 3,985)	90 (2.25%)	3,901 (97.75%)

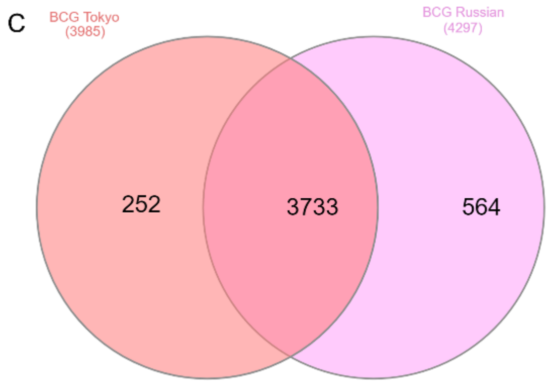

	BCG Tokyo (n = 3,985)	
	Non-homologous	Homologous
BCG Russian (n = 4,297)	252 (6.32%)	3,985 (93.68%)

Figure S1 – (a), (b), (c) Homologous and non-homologous regions between M. tuberculosis H37Rv, early BCG strains and late BCG strains genome sequences.
